# A Novel Combination RNAi toward Warburg Effect by Replacement with miR-145 and Silencing of PTBP1 Induces Apoptotic Cell Death in Bladder Cancer Cells

**DOI:** 10.3390/ijms18010179

**Published:** 2017-01-17

**Authors:** Tomoaki Takai, Yuki Yoshikawa, Teruo Inamoto, Koichiro Minami, Kohei Taniguchi, Nobuhiko Sugito, Yuki Kuranaga, Haruka Shinohara, Minami Kumazaki, Takuya Tsujino, Kiyoshi Takahara, Yuko Ito, Yukihiro Akao, Haruhito Azuma

**Affiliations:** 1 United Graduate School of Drug Discovery and Medical Information Sciences, Gifu University, 1-1 Yanagido, Gifu 501-1193, Japan; uro055@osaka-med.ac.jp (To.T.); uro066@osaka-med.ac.jp (Y.Y.); v3501002@edu.gifu-u.ac.jp (N.S.); v3501001@edu.gifu-u.ac.jp (Y.K.); harukashinohara313@gmail.com (H.S.); t3501001@edu.gifu-u.ac.jp (M.K.); 2 Department of Urology, Osaka Medical College, 2-7 Daigaku-machi, Takatsuki, Osaka 569-8686, Japan; tinamoto@osaka-med.ac.jp (T.I.); uro050@osaka-med.ac.jp (K.M.); uro061@osaka-med.ac.jp (Ta.T.); uro037@osaka-med.ac.jp (K.T.); uro001@osaka-med.ac.jp (H.A.); 3 Department of General and Gastroenterological Surgery, Osaka Medical College, 2-7 Daigaku-machi, Takatsuki, Osaka 569-8686, Japan; sur144@osaka-med.ac.jp; 4 Department of Anatomy and Cell Biology, Division of Life Sciences, Osaka Medical College, 2-7 Daigaku-machi, Takatsuki, Osaka 569-8686, Japan; an1006@osaka-med.ac.jp

**Keywords:** bladder cancer, c-Myc/PTBP1/PKMs axis, combination RNA-interference treatment, miR-145, Warburg effect

## Abstract

Bladder cancer is one of the most difficult malignancies to control. We explored the use of a novel RNA-interference method for a driver oncogene regulating cancer specific energy metabolism by the combination treatment with a small interfering RNA (siRNA) and a microRNA. After transfection of T24 and 253JB-V cells with miR-145 and/or siR-PTBP1, we examined the effects of cell growth and gene expression by performing the trypan blue dye exclusion test, Western blot, Hoechst 33342 staining, reverse transcription polymerase chain reaction (RT-PCR), and electron microscopy. The anti-cancer effects of xenograft model mice with miR-145 and/or siR-PTBP1 were then assessed. The combination treatment induced the deeper and longer growth inhibition and reduced the levels of both mRNA and protein expression of c-Myc and polypyrimidine tract-binding protein 1 (PTBP1) more than each single treatment. Notably, the combination treatment not only impaired the cancer specific energy metabolism by inhibiting c-Myc/PTBP1/PKMs axis but also inactivated MAPK/ERK and PI3K/AKT pathways examined in vitro and in vivo. Furthermore, the combination treatment induced apoptosis or autophagy; but, in some cells, apoptotic cell death was accompanied by autophagy, because the condensation of chromatin and many autophagosomes were coexistent. This combination treatment could be a novel RNA-interference strategy through the systemic silencing of the Warburg effect-promoting driver oncogene *PTBP1* in bladder cancer cells.

## 1. Introduction

Over 73,000 new cases of bladder cancer were identified in the United States in 2012, with approximately 15,000 of these patients dying from the disease during the same year [1]. While the standard of care for muscle invasive bladder cancer (MIBC) is radical cystectomy with bilateral pelvic lymphadenectomy and urinary diversion, [2,3] non-muscle invasive bladder cancer (NMIBC) can be managed with transurethral resection of the bladder tumor and intravesical chemotherapy/immunotherapy [4]. For patients with NMIBC, the issue is to prevent tumor recurrence, which occurs in 50% to 90% of patients within 5 years, and, most importantly, disease progression to muscle invasion, which occurs in up to 20% of patients [5]. On the other hand, patients who are diagnosed as having MIBC also have an unfavorable prognosis, with a 5-year overall and cancer-specific survival period estimated to be approximately 60% [6,7]. Thus, there is a need to identify the driver genes and to develop a more effective therapeutic strategy for bladder cancer. So far, we have been paying attention to the use of RNA medicine such as RNA interference (RNAi), particularly by miR-145, which activates apoptotic pathways and blocks a cancer-specific metabolic pathway responsible for the Warburg effect through the silencing of *c-Myc*. miR-145 is one of the most representative anti-oncomiRs in a variety of cancers, including bladder cancer [8,9,10,11]. We previously reported that miR-145 is downregulated and acts as a tumor suppressor in colon adenomas [8,12], colon cancers [13], gastric cancers [14], chronic lymphocytic leukemias and B-cell lymphomas [15], and several cancer cell lines [16,17,18], especially bladder cancer cells [11,19,20,21]. Notably, miR-145 has a remarkable anti-cancer activity in bladder cancer, as the intravesical delivery of liposome-carrying miR-145 effectively inhibits the tumorigenic phenotypes in a human bladder cancer xenograft mouse model [22]. Recent metabolome analysis indicated that cancer cells exhibit an energy metabolic phenotype characterized by increased glycolysis, regardless of oxygen availability—a phenomenon termed the Warburg effect (Figure S1) [23,24]. The Warburg effect is partly regulated by the expression profiles of pyruvate kinase muscle (PKM) isoforms except pyruvate kinase liver (PKL), which are rate-limiting glycolytic enzymes. PKM has 2 isoforms, PKM1 and PKM2, which are produced by alternative splicing. PKM1 has exon 9 and lacks exon 10, whereas PKM2 has exon 10 and lacks exon 9 [25]. The heterogeneous nuclear ribonucleoprotein (hnRNP) family, which is a main splicer complex including polypyrimidine tract-binding protein 1 (PTBP1), regulates the expression of PKM isoforms [26]. We found that PTBP1 is the splicer responsible for determining the expression profiles of PKM isoforms; and its action leads to PKM2 expression predominantly in cancer cells, which enables the establishment and maintenance of the cancer-specific energy metabolism [27]. Expectedly, we also found the overexpression of PTBP1 in most cancers, including colon, gastric, and bladder. Silencing *PTBP1* by using a small interfering RNA (siRNA) for *PTBP1* (siR-PTBP1) induces a marked growth inhibition with apoptosis and/or autophagy through PKM isoform switching from PKM2 to PKM1, which reflects the metabolic shift from glycolysis to oxidative phosphorylation (OXPHOS) via the tricarboxylic acid cycle [28]. Thus, *PTBP1* is a crucial driver gene that controls the Warburg effect. Despite the availability of many inhibitors for oncogenes, e.g., agents targeting epidermal growth factor receptor (EGFR), vascular endothelial growth factor receptor (VEGFR), or mechanistic target of rapamycin (mTOR) and antibodies, various problems remain, including drug resistance acquisition by genetic mutations and the activation of alternative signaling pathways. Based on such a situation, we decided to explore the silencing of *PTBP1* by siR-PTBP1 and treatment with miR-145, which suppresses the expression systems linked to PTBP1 mainly through the downregulation of c-Myc as an upstream regulator of PTBP1 and inactivation of both MAPK/ERK and PI3K/AKT growth signaling pathways. We concluded that the combination treatment, which aims to block the networks of *PTBP1* expression, exhibited an extreme growth inhibition through perturbation of the Warburg effect and induction of apoptotic cell death.

## 2. Results

### 2.1. Expression of miR-145 Was Extremely Downregulated in Clinical Tumor Samples from Bladder Cancer Patients and Bladder Cancer Cell Lines

**Figure 1:**
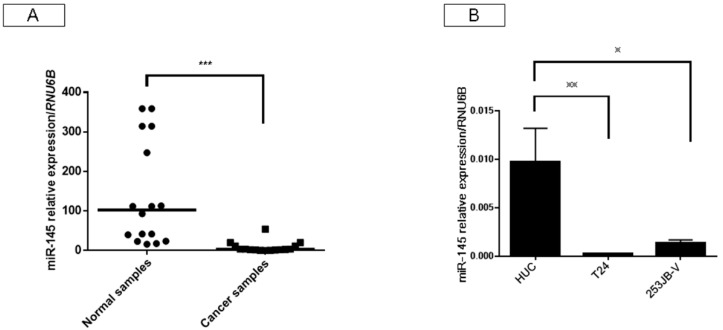
Expression of microRNA (miR)-145 was downregulated in clinical bladder cancer samples and bladder cell lines. (**A**) Relative expression levels of miR-145 in clinical bladder cancer samples; (**B**) Relative expression levels of miR-145 in HUC, T24, and 253JB-V cells. * indicates *p* < 0.05; ** *p* < 0.01; *** *p* < 0.001.

We first examined the expression of miR-145 in bladder cancers and the adjacent normal samples in the same patients, as well as that in various bladder cancer cell lines in this study. As a result, the expression levels of miR-145 in the clinical bladder cancer samples examined by reverse transcription polymerase chain reaction (RT-PCR) using real-time PCR were extremely downregulated compared with those in the normal mucosa (Figure 1A), and also in human bladder cancer T24 and 253JB-V cells (Figure 1B).

### 2.2. Ectopic Expression of miR-145 in Bladder Cancer Cells Induced Apoptosis

**Figure 2:**
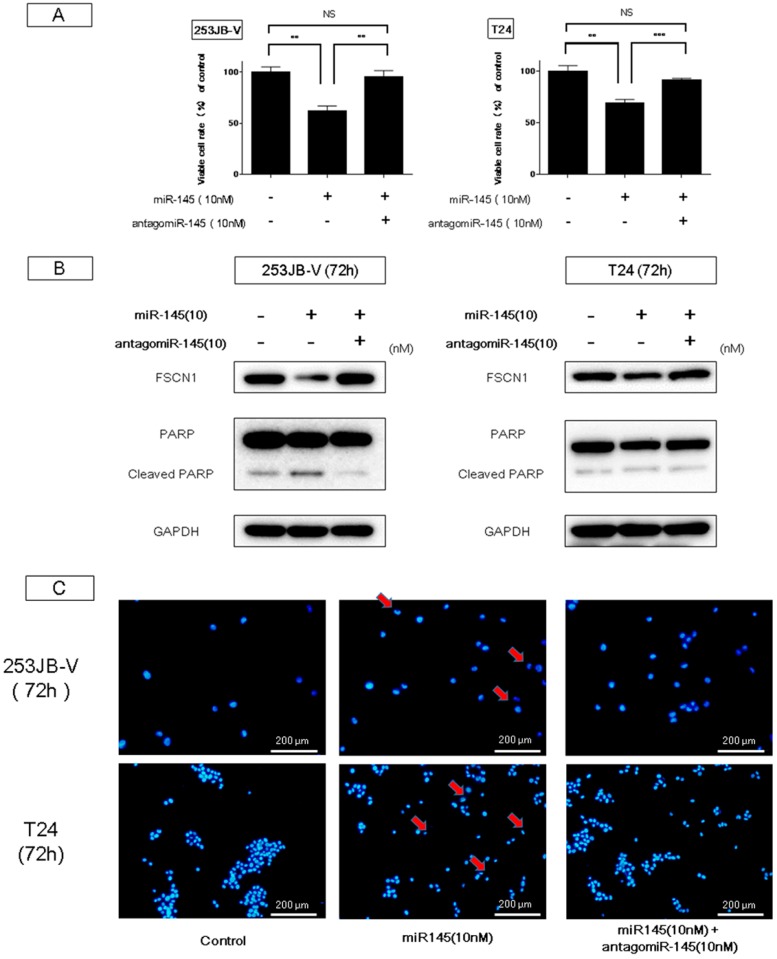

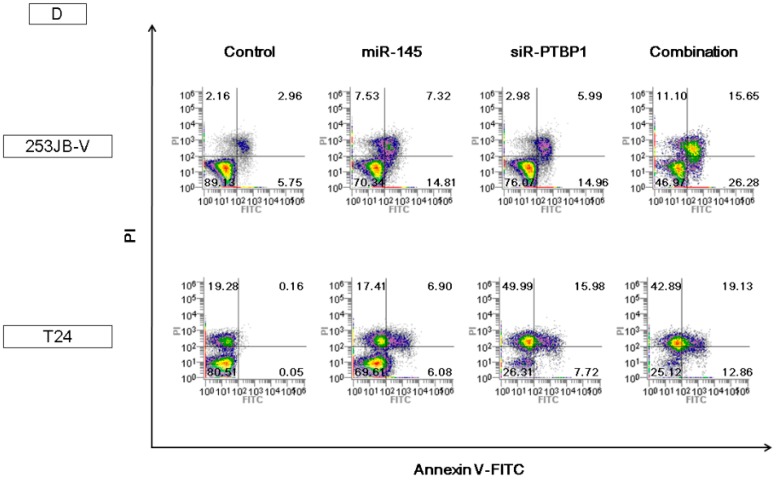
Ectopic expression of miR-145-induced apoptosis in bladder cancer cells. (**A**,**B**) Treatment with antagomiR-145 reversed the growth inhibition and the increased levels of cleaved form of poly (ADP-ribose) polymerase (PARP) elicited by miR-145 in 253JB-V cells; (**C**) Hoechst 33342 staining showed the typical apoptotic features, such as condensed chromatin and nuclear fragmentation indicated by the red arrows, in the miR-145-treated 253JB-V cells; (**D**) Annexin V and propidium iodide (PI) staining evaluated by flow cytometry indicated that combination treatment of miR-145 and siR-PTBP1 induced the apoptosis in 253JB-V and T24 cells compared with control and each single treatment. FITC: fluorescein isothiocyanate. ** indicates *p* < 0.01; *** *p* < 0.001. NS: not significant.

The introduction of miR-145 into bladder cancer 253JB-V and T24 cells induced growth inhibition accompanied by apoptotic cell death, as reported previously [11,22,29]. Western blot analysis indicated the appearance of the cleaved form of poly (ADP-ribose) polymerase (PARP) in 253JB-V and T24 cells transfected with miR-145; and, to the contrary, treatment with antagomiR-145 reversed the growth inhibition and the decreased the level of the cleaved form of PARP elicited by miR-145 introduction (Figure 2A,B). Furthermore, the decreased level of FSCN-1, which is an mRNA typically silenced by miR-145, was also recovered to that in the control sample (Figure 2B). Morphologically, the apoptotic cell number estimated by Hoechst 33342 staining of miR-145-transfected cells was also increased compared with that in the control cells, and also decreased by antagomiR-145 treatment (Figure 2C). Furthermore, results of flow cytometry by annexin V and propidium iodide (PI) staining indicated that combination treatment of ectopic expression of miR-145 and knockdown of *PTBP1* using siR-PTBP1 clearly induced apoptosis in both cell lines compared with each single treatment and control (Figure 2D). Thus, miR-145 acted as an anti-oncomiR in the miR-145-downregulated human bladder cancer cells.

### 2.3. miR-145 Impaired the PTBP1/PKMs Axis, Reducing the Cancer-Specific Energy Metabolism, through Silencing of c-Myc

**Figure 3:**
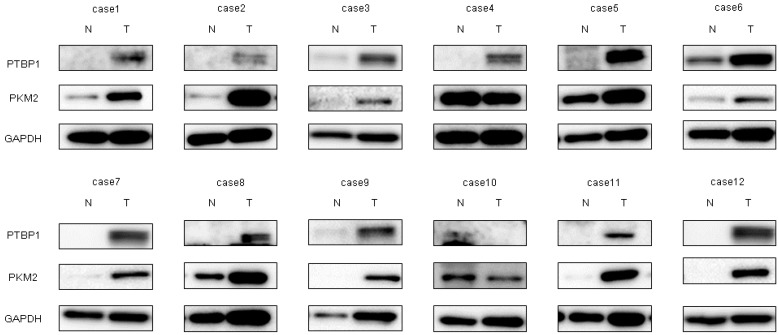
Increased expression levels of polypyrimidine tract-binding protein 1 (PTBP1) and pyruvate kinase muscle isoform 2 (PKM2) were demonstrated in clinical tumor samples from bladder cancer patients. PTBP1 and PKM2 expression in 12 bladder cancer clinical samples as determined by Western blot analysis. Details of the characteristics of the samples are given in Table 1. GAPDH was used as the control.

**Figure 4:**
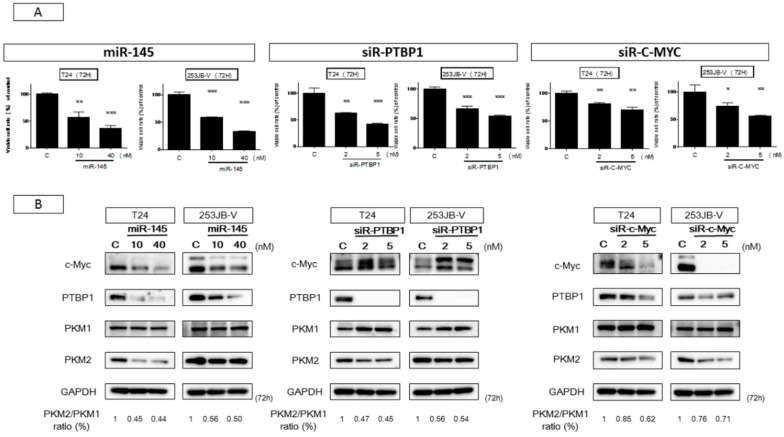
miR-145 impaired PTBP1/PKMs axis in the Warburg effect through downregulation of c-Myc. (**A**) Cell viability after treatment of miR-145 (10 nM, 40 nM), siR-PTBP1 (2 nM, 5 nM), and siR-c-Myc (2 nM, 5 nM) in both cells at 72 h; (**B**) Expression of Warburg effect-related proteins estimated by Western blot analysis after each treatment in T24 and 253JB-V cells, and PKM2/PKM1 ratio calculated based on densitometric values of PKM1 and PKM2. Numbers represent ratios when control values were taken as 1.000. * indicates *p* < 0.05; ** *p* < 0.01; *** *p* < 0.001.

We already reported that the PKM-splicer PTBP1 acts as an oncogene in colon tumors to establish and maintain the cancer specific energy metabolism [30]. To investigate whether PTBP1 in bladder cancer samples was overexpressed, we examined paired samples from 12 bladder cancer patients by Western blot analysis. As shown in Figure 3, the levels of PTBP1 and PKM2 in 11 of the cases were high (91.7%). Details of the characteristics of the samples are given in Table 1. These findings suggested that *PTBP1* acted as an oncogene and that the PTBP1/PKMs axis, in which the PTBP1-splicer promotes the expression of PKM2, [26] functioned to maintain the Warburg effect in bladder cancer. It is a well-known fact that c-Myc, which positively regulates the expression of PTBP1 by acting upstream of it, is a direct target of miR-145 [31]. Based on such findings, we examined the relationship between miR-145 and c-Myc/PTBP1 in the cancer specific energy metabolism. The ectopic expression of miR-145 downregulated c-Myc expression at the translation level and decreased the PKM2/PKM1 ratio in both bladder cancer T24 and 253JB-V cells (Figure 4B), thus suggesting that miR-145 controlled the cancer specific energy metabolism through the c-Myc/PTBP1/PKMs axis. As shown in Figure 4A,B, the ectopic expression of miR-145 significantly downregulated PTBP1 through knockdown of *c-Myc*, resulting in growth inhibition in a concentration-dependent manner in both cells. Importantly, the introduction of either miR-145, siR-PTBP1, or siR-c-Myc induced growth inhibition through the switching from PKM2 to PKM1, resulting in a reduced PKM2/PKM1 ratio (Figure 4A,B). These results strongly suggest that c-Myc and PTBP1 positively regulated the cancer-specific energy metabolism through the c-Myc/PTBP1/PKMs axis. Thus, miR-145 negatively affected the Warburg effect through the downregulation of c-Myc, which upregulated PTBP1 in these cell lines.

### 2.4. Increased Expression of miR-145 Combined with Knockdown of PTBP1 Contributed to the Greater and Longer Growth Suppression Compared with Each Single Treatment

**Figure 5:**
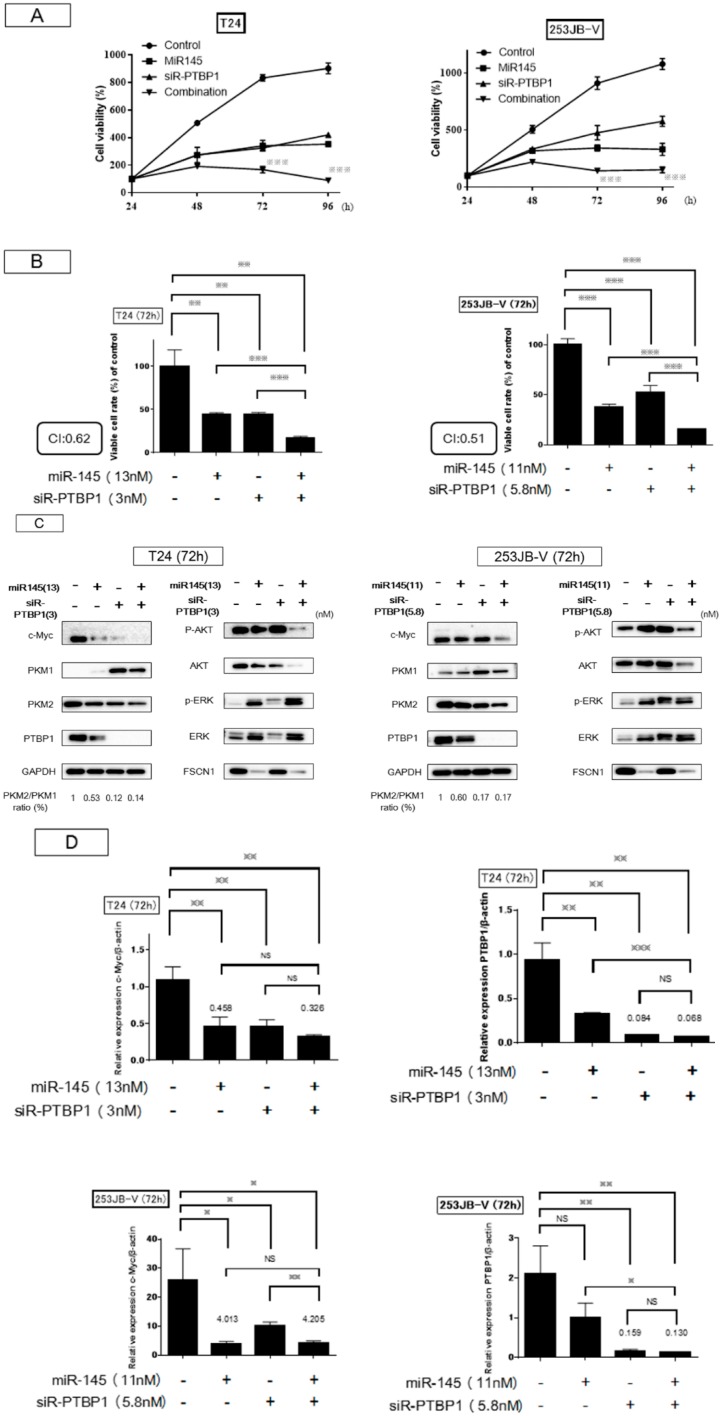

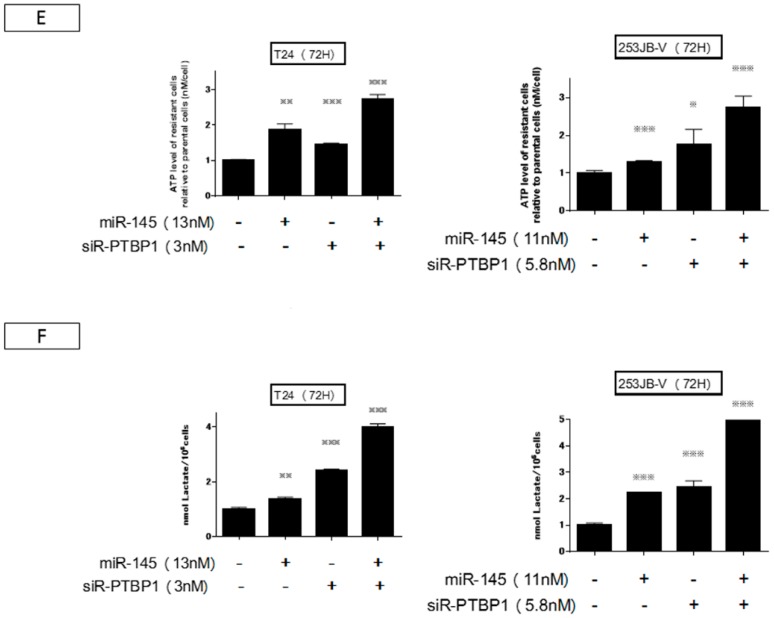
Combination treatment contributed to the greater and longer growth inhibition compared with each single-treatment. (**A**,**B**) The graph showed a time-course and at 72 h of cell growth after treatment with miR-145 and/or siR-PTBP1. Combination treatment inhibited cell growth greater and deeper than single-treatment. CI: Combination index. The combination index theorem of Chou-Talalay was used as a quantitative definition for additive (CI = 1) and synergistic (CI < 1) effects of drug combinations. (**C**,**D**) The expression of growth and Warburg effect-related proteins examined by Western blot analysis and mRNA levels of c-Myc and PTBP1 examined by RT-PCR in T24 and 253JB-V cells at 72 h after miR-145 and/or siR-PTBP1 were shown. (**E**,**F**) Intracellular ATP and lactic acid levels were measured at 72 h after the transfection of T24 and 253JB-V cells with miR-145 and/or PTBP1. * indicates *p* < 0.05; ** *p* < 0.01; *** *p* < 0.001.

Next, we examined the effect on cell growth by the combination treatment at the half maximal inhibitory concentration (IC50) of miR-145 as a replacement treatment and knockdown of *PTBP1* by siR-PTBP1 in both cancer cells. Basically, with each single treatment the viable cell number increased up to 96 h. However, the combination treatment induced greater and longer growth inhibition compared with each single treatment: after 48 h, the viable cell number decreased up to 96 h (Figure 5A). The combination index was 0.6 and 0.5 for T24 and 253JB-V cells, respectively (Figure 5A,B). Western blot analysis at 72 h indicated that treatment with either agent alone reduced the expression level of PTBP1 and modulated the cancer specific energy metabolism through switching of PKM isoform expression from PKM2 to PKM1, thus resulting in a reduction in the PKM2/PKM1 ratio (Figure 5C). The ratio was almost the same between the cases of miR-145 alone and the combination treatment. Importantly, the combination treatment markedly reduced the protein expression levels of c-Myc and PTBP1 consistently. Furthermore, this treatment of T24 cells caused a marked reduction in the level of *c-Myc* mRNA, leading to a further reduction in the *PTBP1* mRNA level compared with the level found with the siR-PTBP1 single treatment (Figure 5D). The decrease in the *c-Myc* mRNA level by the combination treatment resulted in the downregulation of *PTBP1* even in 253JB-V cells (Figure 5D). These results suggested that the combination treatment could not only decrease the levels of c-Myc and PTBP1 protein, but also suppress the expression system such as transcription and/or network linking c-Myc/PTBP1/PKMs axis with *PTBP1* expression probably by the downregulation of both *c-Myc* mRNA and protein expression. We next examined the intracellular levels of lactic acid and ATP to investigate metabolic changes as a consequence of suppression of the c-Myc/PTBP1/PKMs axis operating in the cancer specific energy metabolism and of switching PKM isoforms from PKM2 to PKM1 by each treatment (Figure 5E,F). The data on the ATP levels reflected the suppression of glycolysis and, in turn, the promotion of OXPHOS after the switching of PKM isoforms (Figure 5E). Especially in the case of the combination treatment, the increment was prominent. On the other hand, an increased level of lactic acid was found with miR-145 or combination treatment (Figure 5F), which may indicate the gluconeogenesis from lactic acid in response to the perturbation of the cancer-specific energy metabolism.

### 2.5. The Combination Treatment of Ectopic Expression of miR-145 and Knockdown of PTBP1 Induced Apoptosis and Autophagy

**Figure 6:**
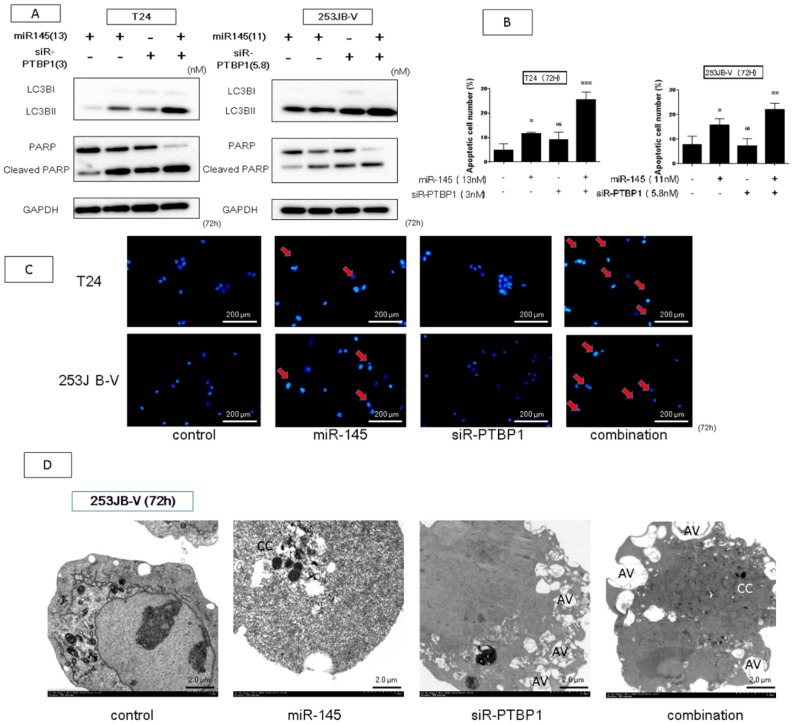
The combination treatment induces apoptosis and autophagy. (**A**,**B**) Hoechst 33342 staining at 72 h after transfection of miR-145 and/or siR-PTBP1 with T24 and 253JB-V cells. Apoptotic cell is indicated by a red arrow. (**C**) To validate the apoptosis-inducing effect on each treatment, Western blot analysis was performed. Combination treatment increased the level of cleaved form of PARP and led to transition from LC3BI to LC3BII. (**D**) Morphological study by using electron microscopy was performed at 72 h after transfection with each treatment in 253JB-V cells. AV: autophagosomes, CC: condensation of the chromatin. * indicates *p* < 0.05; ** *p* < 0.01; *** *p* < 0.001.

Earlier we reported that ectopic expression of miR-145 induces apoptosis and that knockdown of *PTBP1* leads to autophagy and/or apoptosis [11,28]. Biochemically, the ectopic expression of miR-145 resulted in the appearance of the cleaved form of PARP and knockdown of *PTBP1* induced the transition of the LC3BI to LC3BII in both types of cancer cells (Figure 6A). Hoechst 33342 nuclear staining showed the typical apoptotic features, such as condensed chromatin and nuclear fragmentation in the miR-145-treated and combination-treated cells (Figure 6B,C). On the other hand, we found that the combination treatment induced both apoptosis and autophagy simultaneously, as examined by Western blot analysis, because of the presence of the cleaved form of PARP and transition of LC3BI to LC3BII (Figure 6A). Interestingly, the morphological study by electron microscopy indicated typical apoptotic cells accompanied by autophagic appearance in the combination-treated 253JB-V cells, because the cells showed condensed chromatin and many autophagosomes in the same cells. These results suggested that the combination treatment not only induced apoptosis but also autophagy-like cells that proceeded to undergo apoptotic cell death (Figure 6D).

### 2.6. Antitumor Effect of miR-145 and/or siR-PTBP1 on 253JB-V Cell Xenografted Tumors in Nude Mice

**Figure 7:**
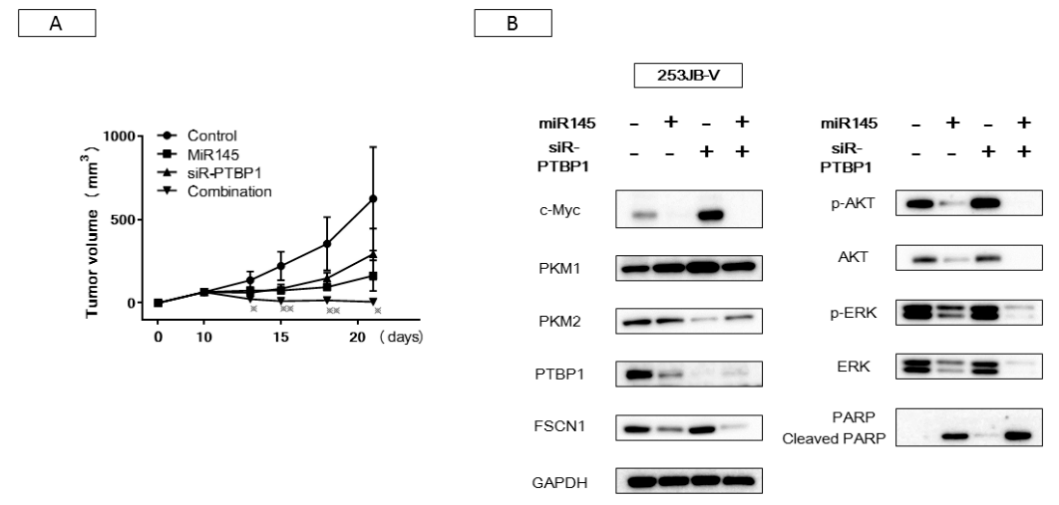
Antitumor effect of miR-145 or/and siR-PTBP1 on 253JB-V cells xenografted tumors induced in nude mice. (**A**) The graph shows time-course of tumor size in mice injected with each treatment. * indicated the significant in the tumor sizes in combination injection compared with each single-injection. (**B**) Expression levels of growth and Warburg effect-related proteins in each tumor tissue examined by Western blot analysis. * indicates *p* < 0.05; ** *p* < 0.01.

In order to examine the effect of miR-145 and/or siR-PTBP1 on a xenograft mouse model, we inoculated 253JB-V cells subcutaneously into nude mice. At 10 days after the inoculation, we injected a solution containing control-miR, miR-145, siR-PTBP1, or the combination of miR-145 and siR-PTBP1 into the tumors. As a result, a significant suppression of tumor growth was observed in either single-injection group, miR-145 or siR-PTBP1. Notably, the combination injection exhibited a remarkable reduction in tumor size, as shown in Figure 7A. Western blot analysis of protein samples from the treated tumors gave results similar to those obtained in vitro (Figure 7B). These findings indicated that the combination treatment exerted its antitumor effect by perturbing networks of growth signaling pathways including PI3K/AKT, MAPK/ERK, and the Warburg effect-related c-Myc/PTBP1/PKMs axis, even in vivo.

## 3. Discussion

**Scheme 1:**
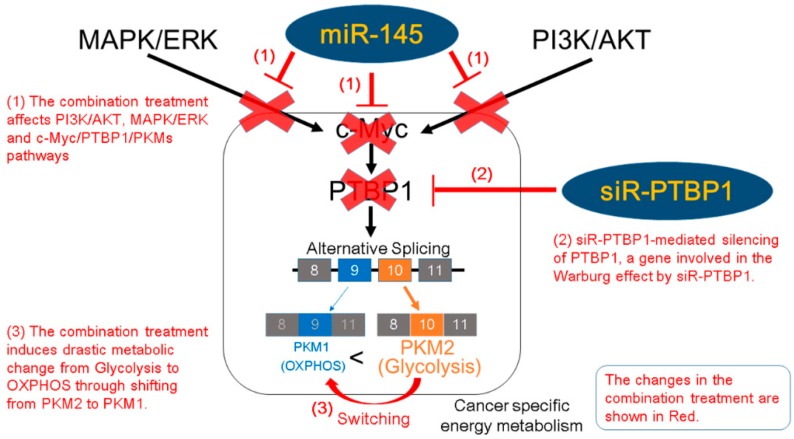
Schematic representation of the effects of the combination RNA interference (RNAi) treatment on silencing *PTBP1*, a gene involved in the cancer specific energy metabolism. OXPHOS: oxidative phosphorylation.

Although we have many kinds of medicine for cancers, including antineoplastic agents and molecule-targeted low-molecular agents and antibodies, the acquisition of drug resistance and presence of residual cancer stem cells are most important problems. In this study, we aimed to develop a novel strategy of combination RNAi treatment for perturbation of the cancer specific energy metabolism to avoid these problems. Earlier, we found that PTBP1, a major splicer of the *PKM* gene, is a gene responsible for maintenance of the cancer specific energy metabolism and that it is frequently overexpressed in clinical tumor samples [30]. In the current study, we investigated whether the c-Myc/PTBP1/PKMs axis was crucial for the Warburg effect, in which *PTBP1* is oncogenic and an important driver gene in many kinds of cancers, possibly in cancer stem cells. To explore this possibility, we examined the effect of combination RNAi treatment by using miR-145, which can silence *c-Myc* expression and siR-PTBP1 to negate the action of the driver gene *PTBP1* in cancer pathogenesis. Expectedly, the combination treatment induced an extreme reduction in the *PTBP1* mRNA and protein levels through downregulation of *c-Myc*, which is known to positively regulate *PTBP1* gene expression. Resultantly, the combination treatment caused a PKM isoform switching from PKM2 to PKM1 and finally severe apoptotic cell death. We consider that some of the autophagic cells transfected with siR-PTBP1 and miR-145 underwent apoptotic cell death in the late phase, which was shown by the results of the biochemical and electron microscopic studies. Although one medicine for a driver oncogene activated alternative signaling pathways, the combination treatment including miR-145, which affects various oncogenes, restrained the alternative signaling pathways including MAPK/ERK, PI3K/AKT, and *c-Myc*, which are necessary to maintain the cancer specific energy metabolism. Thus, the combination RNAi treatment systematically downregulated *PTBP1* expression, which would not be achieved by siR-PTBP1 treatment alone. In the case of human colon cancer DLD-1 cells, the combination treatment reduced the expression of c-Myc and PTBP1 at both mRNA and protein levels for a longer time than seen with the T24 or 253JB-V cells, which led to profoundly greater inhibition. There was a synergy effect even on the colon cancer cells, because the combination index for the DLD-1 cells was 0.95 (less than 1.0) (Figure S2). These results suggest that this combination treatment using ectopic expression of miR-145 and knockdown of *PTBP1* would also cause significant growth inhibition even in other cancers. We consider that the combination of siRNA for the driver gene *PTBP1* and the miR-145 silencing of the plural genes associated with the networks related to *PTBP1* expression, such as *c-Myc* and PI3K/AKT signaling molecules, could avoid drug resistance of various kinds of cancers (Scheme 1). For intermediate- and high-risk non-muscle-invasive bladder cancer after transurethral resection of a bladder tumor (TUR-BT), intravesical bacillus Calmette–Guérin (BCG) is currently considered the first-line treatment. However, approximately 50% of patients treated with BCG experience intravesical disease recurrence and/or progression, commonly referred to as BCG failure. Hence, we should find alternative therapy; and the results described in our current study showed that the combined RNAi medicine could represent an alternative to BCG therapy. However, there are well-known problems, such as degradation by serum RNase, in using RNA medicine. We previously found that intravesical instillation of liposome-encapsulated miR-145 after emptying the residual urine from the bladder has an antitumor effect in a human bladder cancer xenograft mouse model [22]. To further enhance the antitumor effects of miR-145, we have developed a novel synthetic version of it. This synthetic miR-145 showed greater growth inhibition compared with any commercially supplied miR-145s for use in in vitro experiments. In the future, we plan to perform a double-blind comparative study on BCG therapy and this synthetic miR-145 in vivo.

## 4. Materials and Methods

### 4.1. Patients and Samples

All human samples were obtained from patients who had undergone biopsy or surgery for resection at Osaka Medical College Hospital (Takatsuki, Osaka, Japan). Informed consent in writing was obtained from each patient. 

The consent and this study were reviewed and approved by the University hospital medical information network center (approval number: R000027312; date of approval: 8 April 2016), in accordance with the tenets of the Declaration of Helsinki. Twelve patients with previously untreated (or recently diagnosed) bladder cancer were selected. The distribution according to other clinical parameters is shown in Table 1. Under a pathologist’s supervision, all tissue sample pairs were collected from surgically resected tissues, with these paired samples being from the primary tumor and its adjacent non-tumor tissue in the same patient. These paired samples were examined by Western blot analysis and real-time reverse transcription PCR.

### 4.2. Cell Culture and Cell Viability

All cell lines were obtained from the JCRB (Japanese Collection of Research Bioresources) Cell Bank. They were cultured in RPMI-1640 medium supplemented with 10% (*v*/*v*) heat-inactivated fetal bovine serum (FBS, Sigma-Aldrich Co., St. Louis, MO, USA) and 2 mm l-glutamine under an atmosphere of 95% air and 5% CO_2_ at 37 °C. The number of viable cells was determined by performing the trypan-blue dye-exclusion test.

### 4.3. Transfection Experiments

T24 cells or 253JB-V cells were seeded into 6-well plates at a concentration of 0.5 × 10^5^ per well (10%–30% confluence) on the day before the transfection. The mature type of miR-145 (mirVanaTM miRNA mimic; Ambion, Foster City, CA, USA) was used for the transfection of the cells, which was achieved by using cationic liposomes, Lipofectamine RNAiMAX (Invitrogen, Carlsbad, CA, USA), according to the manufacturer’s Lipofection protocol. The nonspecific control miRNA (HSS, Hokkaido, Japan) sequence was 5′-GUAGGAGUAGUGAAAGGCC-3′, which was used as a control for nonspecific effects [8]. In single treatment or combination treatment experiments, the sequence of the mature type of miR-145 used in this study was 5′-GUCCAGUUUUCCCAGGAAUCCCUU-3′; and that of siR-PTBP1, 5′-AUCUCUGGUCUGCUAAGGUCACUUC-3′. The effects manifested by the introduction of miR-145 and/or siR-PTBP1 into the cells were assessed at 72 h after the transfection. We used the same doses of Lipofectamine RNAiMAX in all transfection experiments.

### 4.4. Western Blot Analysis

Protein extraction and Western blotting analysis were performed as described in previous reports [29,32]. The following primary antibodies were used: antibodies against c-Myc, PTBP1, p-AKT, AKT, p-ERK, ERK, PARP, and GAPDH (Cell Signaling Technology, Inc., Danvers, MA, USA), PKM1, and PKM2 (Novus Biologicals, Littleton, CO, USA) and FSCN1 (Abcam, Cambridge, UK). HRP-conjugated goat anti-rabbit and horse anti-mouse IgG (Cell Signaling Technology) were used as secondary antibodies. GAPDH served as an internal control.

### 4.5. Real-Time Reverse Transcription PCR

Total RNA was isolated from cultured cells or tumor tissues by using a NucleoSpin miRNA isolation kit (TaKaRa, Otsu, Japan). RNA concentrations and purity were assessed by UV spectrophotometry. RNA integrity was checked by formaldehyde gel electrophoresis. To determine the expression levels of miR-145, we conducted quantitative RT-PCR (qRT-PCR) by using TaqMan MicroRNA Assays (Applied Biosystems, Foster City, CA, USA) and THUNDERBIRD Probe qPCR Mix (TOYOBO Co., LTD., Osaka, Japan) according to the manufacturer’s protocol. RNU6B was used as an internal control. For determination of the expression levels of *c-Myc*, *PTBP1*, and *β-actin* mRNAs, total RNA was reverse-transcribed with a PrimeScriptH RT reagent Kit (TaKaRa). RT-PCR was then performed with primers specific for them by using THUNDERBIRD SYBR qPCR Mix (TOYOBO). The primers for *c-Myc*, *PTBP1*, and *β-actin* were the following: c-Myc-sense, 5′-TTCGGGTAGTGGAAAACCAG-3′, and c-Myc-antisense, 5′-CAGCAGCTCGAATTTCTTCC-3′; PTBP1-sense, 5′-ATCAGGCCTTCATCGAGATGCACA-3′, and PTBP1-antisense, 5′-TGTCTTGAGCTCCTTGTGGTTGGA-3′; β-actin-sense, 5′-TGACGGGGTCACCCACACTGTGCCCATCTA-3′, and β-actin-antisense, 5′-CTAGAAGCATTTGCGGTGGACGATGGAGGG-3′. *β-actin* was used as an internal control. The relative expression levels were calculated by the ΔΔ*C*_t_ method.

### 4.6. Hoechst 33342 Staining

T24 cells or 253JB-V cells were collected at 72 h after transfection. The details of the experimental protocol were given in a previous report [28]. The number of apoptotic cells among 400 cells was counted.

### 4.7. Apoptosis Assay Using Flow Cytometry

Cell apoptosis was analyzed using a propidium iodide (PI)/annexin V-fluorescein isothiocyanate (FITC) double staining cell apoptosis detection kit (Molecular Probes, Eugene, OR, USA), according to the manufacturer’s protocol. A total of 5.0 × 10^5^ cells (253JB-V or T24) were seeded into a 6-well plate and transfected with miR-145 and/or siR-PTBP1 or control microRNA in 253JB-V and T24 cells. The total cells at 72 h after the transfection were collected and stained with annexin V/PI, and the percentage of apoptotic and viable cells was determined by flow cytometry EC800 cell analyzer (Sony Corp, Minato-ku, Tokyo, Japan).

### 4.8. Lactate Assay

Cells were incubated with miR-145 and/or PTBP1 for 72 h. Intracellular l-lactate was extracted by using an l-lactate Assay kit (Cayman Chemical Company, Ann Arbor, MI, USA). l-lactate production was measured with a Lactate Colorimetric/Fluorometric Assay kit (Biovision, Milpitas, CA, USA) according to the manufacturer's instructions. Lactate production was normalized to cell numbers.

### 4.9. ATP Assay

To measure the ATP levels before the cells were committed to programmed cell death, we incubated them with miR-145 and/or PTBP1 for 72 h. ATP production was measured with an ATP Determination Kit (A22066; Invitrogen) according to the manufacturer’s instructions. ATP production was normalized to cell numbers.

### 4.10. In Vivo Xenograft Model

Animal experimental protocols were approved by the Committee for Animal Research and Welfare of Gifu University. BALB/cSLC-nu/nu (nude) mice were obtained from Japan SLC (Hamamatsu, Japan). Human bladder cancer 253JB-V cells were inoculated at 5.0 × 10^6^ cells/100 μL per site into the back of each mouse. The inoculation day was set as day 0. At 10 days after inoculation, we confirmed the engraftment of the tumors. After control miRNA, miR145 and/or siR-PTBP1 (0.2 nmol per 1 administration) in 50 μL of Opti-MEM had been incubated with 1 μL of Lipofectamine RNAiMAX, the mixture was injected into the tumor every 72 h. The tumor volume was calculated by the following formula: 0.5236 L1 × (L2)^2^, where L1 is the long axis and L2 is the short axis of the tumor. Animal experiments in this study were performed in compliance with the guidelines of the Institute for Laboratory Animal Research of Gifu University (approval number: 11021, approval date: 10 February 2016), and with the UKCCCR Guidelines for the Welfare of Animals Used for Experimental Neoplasia. Yukihiro Akao was named in the approved experiment.

### 4.11. Statistics

Each examination was performed in triplicate. Statistical differences between clinicopathologic parameters and the miR-145 level of tumor samples were evaluated by using Pearson’s χ^2^-test or Fisher’s exact test, unless otherwise specified. For in vitro and in vivo experiments, statistical significances of differences were evaluated by performing the two-sided Student’s *t*-test. The values were presented as the mean ± standard deviation. A *p*-value < 0.05 was considered to be statistically significant.

## 5. Conclusions

We described here the first novel combination RNAi treatment using ectopic expression of miR-145 and knockdown of *PTBP1*. We found that this combination treatment had an antitumor effect on the cancer-specific pathways including PI3K/AKT, MAPK/ERK, and c-Myc/PTBP1/PKMs involved in the Warburg effect in bladder cancer. We propose that this combination treatment has the potential to become a successful therapeutic strategy to overcome the drug resistance of bladder cancer cells.

## Figures and Tables

**Table 1:** Clinicopathological patient features.

Case	Age	Sex	Size (cm)	Grade	T Stage	miR-145
**1**	63	M	4	2	pTa	D
**2**	68	M	1	1	pTa	D
**3**	79	F	2	2	pT1	D
**4**	49	M	3	3	pT1	D
**5**	74	F	4	3	pT2	D
**6**	71	M	3	3	pT1	D
**7**	67	M	5	2	pT1	D
**8**	84	F	3	1	pT1	D
**9**	75	M	3	1	pTa	D
**10**	79	F	2	2	pTa	D
**11**	60	M	4	1	pTa	D
**12**	86	M	20	3	pT1	D

M: male; F: female; D: downregulation of miR-145 relative ratio.

## Supplementary Materials

Supplementary materials can be found at www.mdpi.com/1422-0067/18/1/179/s1.
