# Supplementary material for: A Novel Combination RNAi toward Warburg Effect by Replacement with miR-145 and Silencing of PTBP1 Induces Apoptotic Cell Death in Bladder Cancer Cells
Source: Int J Mol Sci. 2017 Jan 17;18(1):179. doi: 10.3390/ijms18010179 (PMC5297811; doi:10.3390/ijms18010179)
Supplement: Supplementary file 1 [file ijms-18-00179-s001.pdf]

Int. J. Mol. Sci. **2017**, *18*, 179: doi:10.3390/ijms18010179

Supplementary Materials: A Novel Combination RNAi toward Warburg Effect by Replacement with miR-145 and Silencing of PTBP1 Induces Apoptotic Cell Death in Bladder Cancer Cells

Tomoaki Takai, Yuki Yoshikawa, Teruo Inamoto, Koichiro Minami, Kohei Taniguchi, Nobuhiko Sugito, Yuki Kuranaga, Haruka Shinohara, Minami Kumazaki, Takuya Tsujino, Kiyoshi Takahara, Yuko Ito, Yukihiro Akao and Haruhito Azuma

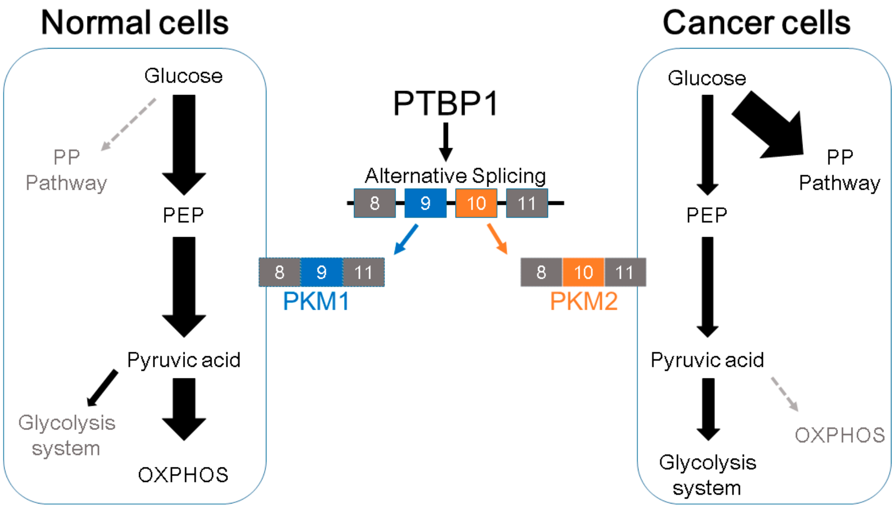

Figure S1. Schematic of metabolic pathway in the normal cells and cancer cells. PTBP1: polypyrimidine tract-binding protein 1; PEP: phosphoenol pyruvate; PP: pentose phosphate; OXPHOS: oxidative phosphorylation; PKM: pyruvate kinase muscle.

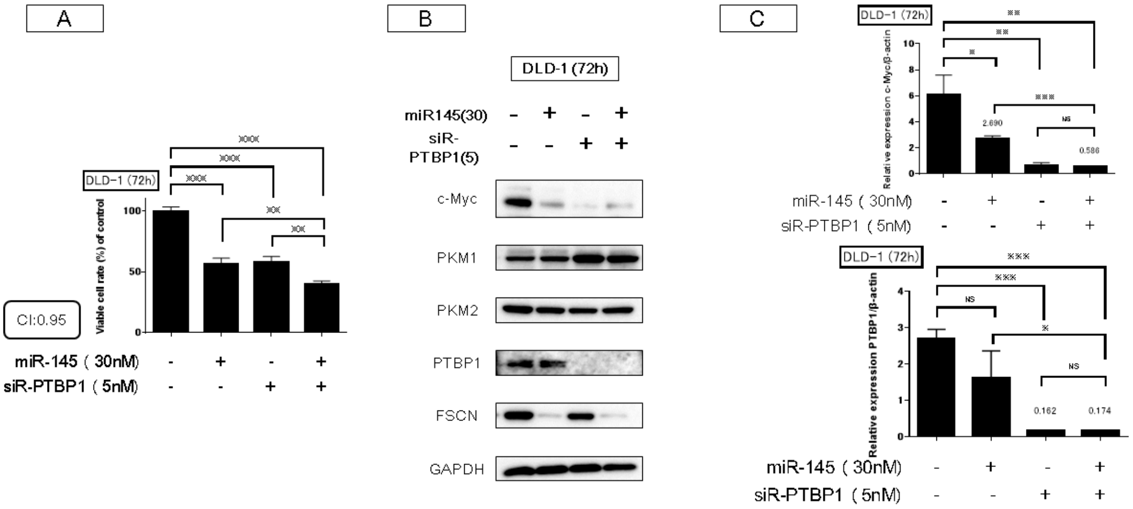

Figure S2. Antitumor effect of miR-145 or/and siR-PTBP1 on colon cancer DLD-1 cells. (**A**) Effects of each treatment on cell viability at 72 h after transfection of DLD-1 cells with miR-145 and/or siR-PTBP1. CI: combination index. (**B**,**C**) Expression level of Warburg effect-related proteins examined by Western blot analysis and mRNA levels of c-Myc and PTBP1 examined by reverse transcription polymerase chain reaction (RT-PCR) at 72 h after the transfection. * indicates $p < 0.05$; ** $p < 0.01$; *** $p < 0.001$.
